# Supplementary material for: The evolutionary path of chemosensory and flagellar macromolecular machines in Campylobacterota
Source: PLoS Genet. 2022 Jul 14;18(7):e1010316. doi: 10.1371/journal.pgen.1010316 (PMC9321776; doi:10.1371/journal.pgen.1010316)
Supplement: S1 Fig — (PDF) [file pgen.1010316.s001.pdf]

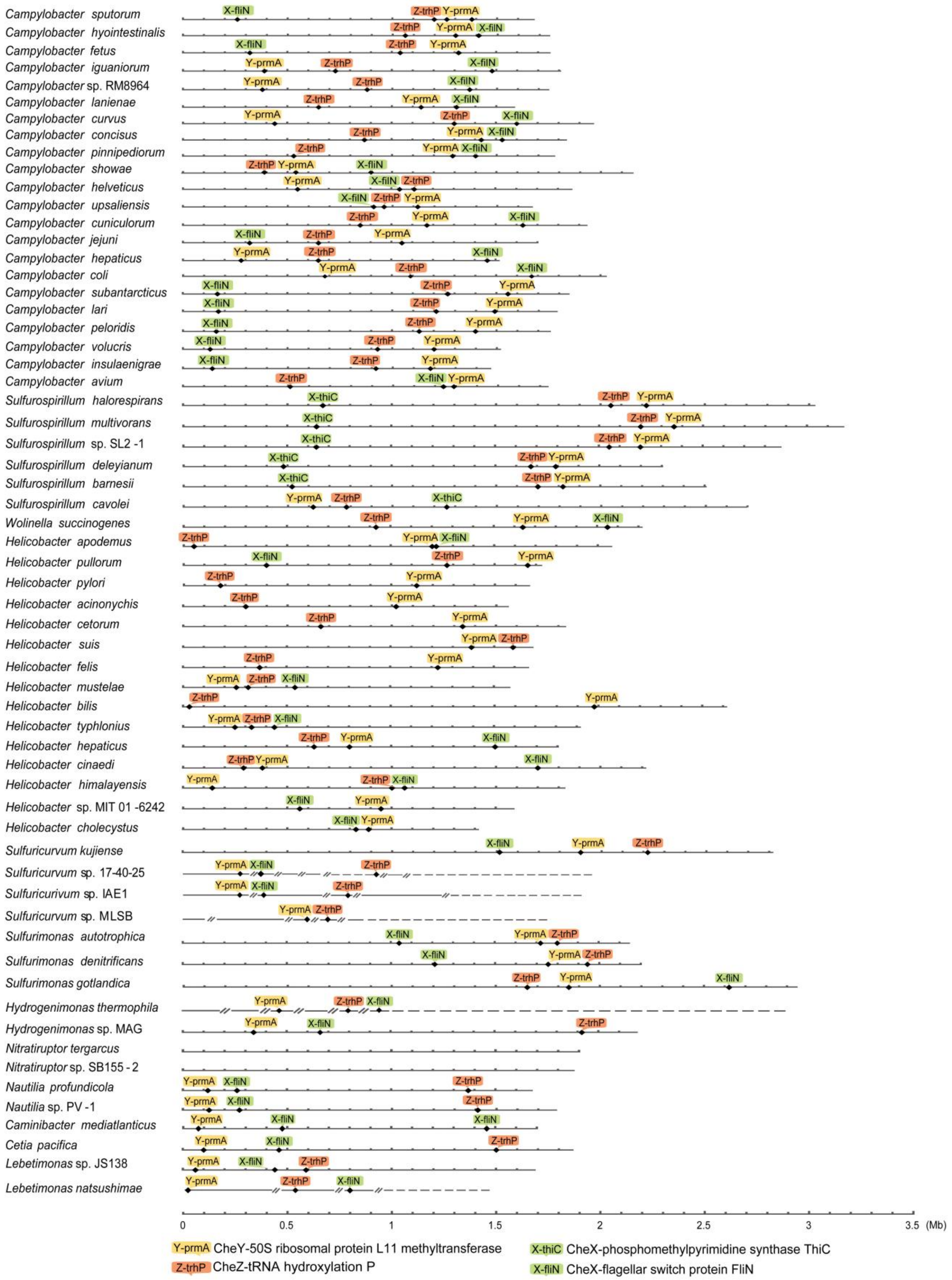

S1 Fig. The conserved gene order of F3 chemosensory class genes *cheX*, *cheY*, and *cheZ*. Abbreviations: X-*fliN*, CheX-flagellar switch protein FliN; Y-*prmA*, CheY-50S ribosomal protein L11 methyltransferase; Z-*trhP*, CheZ-tRNA hydroxylation P.
