# Supplementary material for: The evolutionary path of chemosensory and flagellar macromolecular machines in Campylobacterota
Source: PLoS Genet. 2022 Jul 14;18(7):e1010316. doi: 10.1371/journal.pgen.1010316 (PMC9321776; doi:10.1371/journal.pgen.1010316)
Supplement: S4 Fig — (PDF) [file pgen.1010316.s004.pdf]

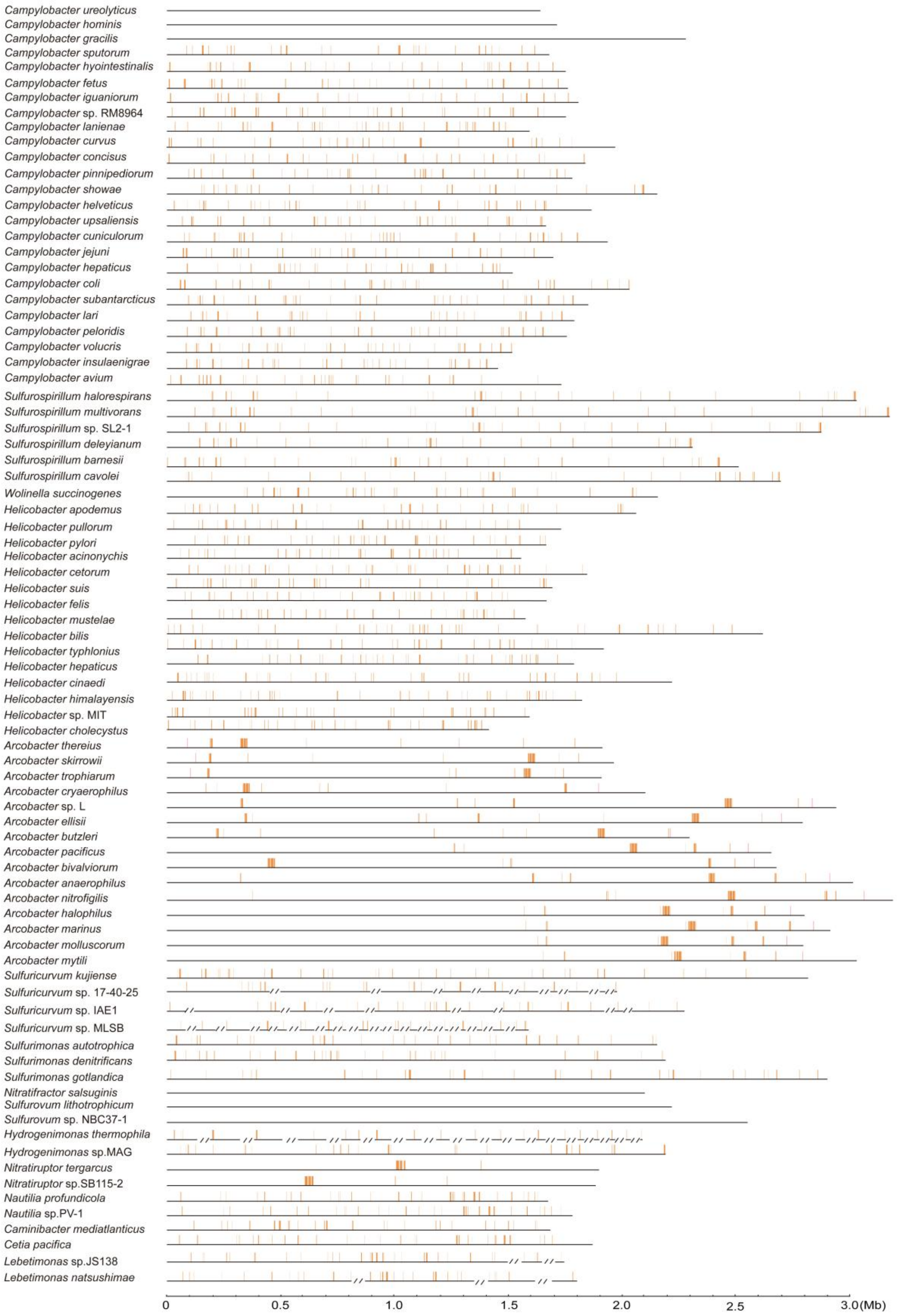

S4 Fig. Genomic distribution of flagellar genes in *Campylobacterota* species. The linearized genomes are depicted as scale lines and flagellar genes as the ochre stripes.
