# Supplementary material for: The evolutionary path of chemosensory and flagellar macromolecular machines in Campylobacterota
Source: PLoS Genet. 2022 Jul 14;18(7):e1010316. doi: 10.1371/journal.pgen.1010316 (PMC9321776; doi:10.1371/journal.pgen.1010316)
Supplement: S5 Fig — (PDF) [file pgen.1010316.s005.pdf]

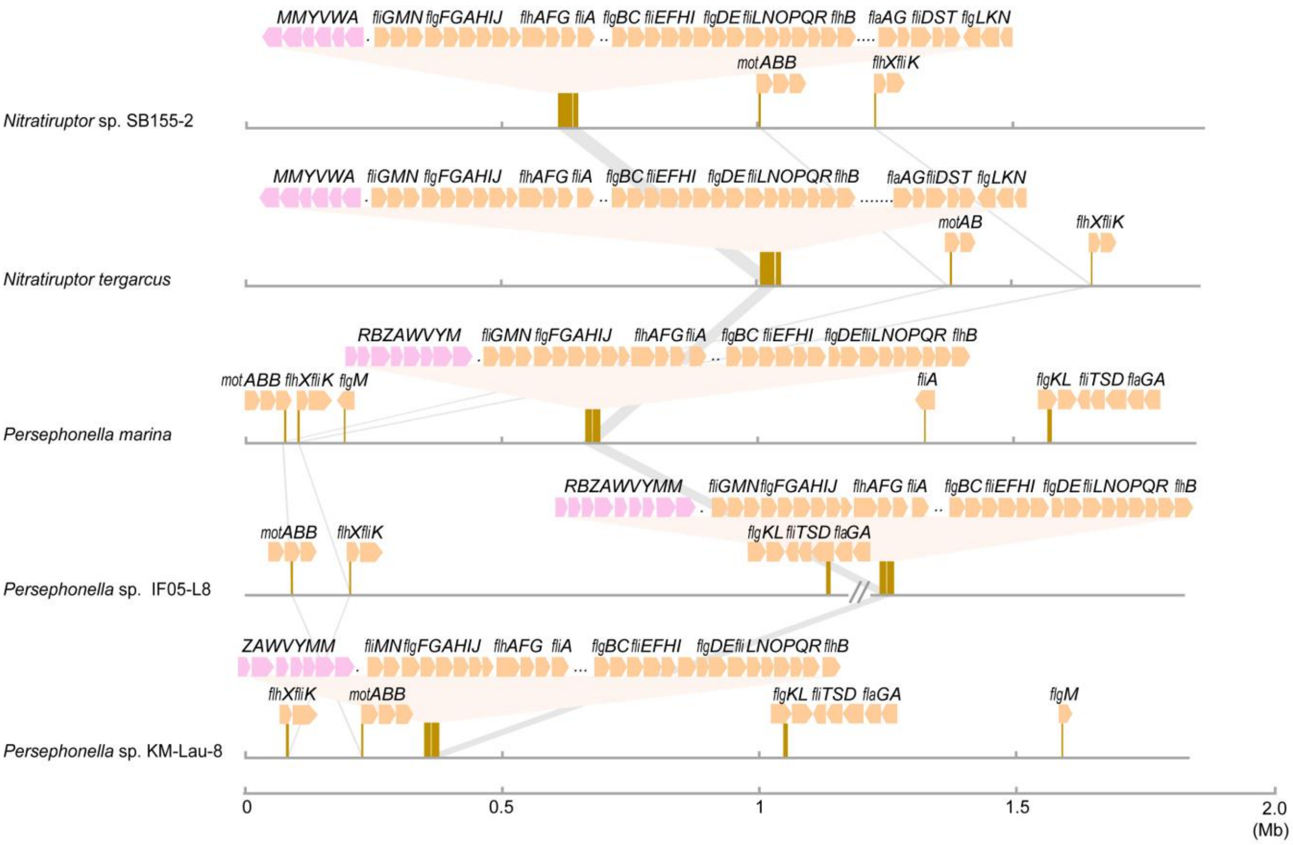

S5 Fig. The gene order of F14 chemosensory class and flagellar locus in species of *Nitratiruptor* (*Campylobacterota*) and *Persephonella* (*Aquificota*). Ochre strips represent flagellar genes on the linearized genomes; the order of flagellar (orange arrow) and chemosensory gene (pink arrow) are depicted above; the grey lines link the same gene cluster from different genomes.
