# Supplementary material for: The evolutionary path of chemosensory and flagellar macromolecular machines in Campylobacterota
Source: PLoS Genet. 2022 Jul 14;18(7):e1010316. doi: 10.1371/journal.pgen.1010316 (PMC9321776; doi:10.1371/journal.pgen.1010316)
Supplement: S6 Fig — (A) Ternary comparison of flagellar gene distribution of six Arcobacter species with deep-branching species (Lebetimonas sp. JS138) and a close relative (Sulfurimonas gotlandica GD1). (B) The gene order of two big flagellar gene clusters (orange arrow) in Arcobacter species. (PDF) [file pgen.1010316.s006.pdf]

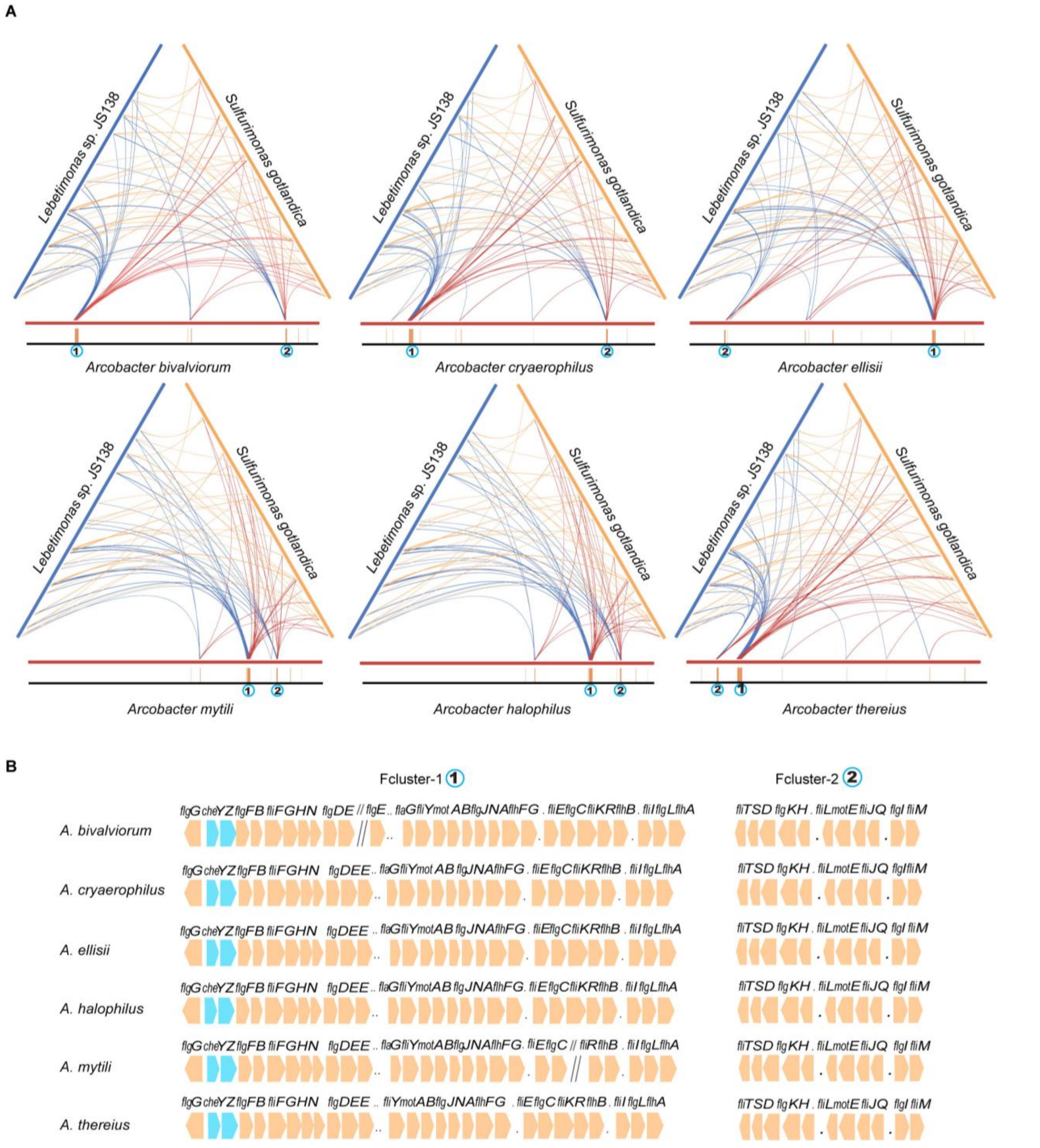

S6 Fig. (A) Ternary comparison of flagellar gene distribution of six *Arcobacter* species with deep-branching species (*Lebetimonas* sp. JS138) and a close relative (*Sulfurimonas gotlandica* GD1). Below each ternary comparison, the flagellar genes of the *Arcobacter* species are depicted by ochre strips on linearized genomes, and the numerical symbols demonstrate two big flagellar gene clusters: Fcluster-1 and Fcluster-2. **(B)** The gene order of two big flagellar gene clusters (orange arrow) in *Arcobacter* species. The blue arrows represent two chemosensory genes *cheYZ* located inside of Fcluster-1.
