# Supplementary figures and images for: The evolutionary path of chemosensory and flagellar macromolecular machines in Campylobacterota
Source: PLoS Genet. 2022 Jul 14;18(7):e1010316. doi: 10.1371/journal.pgen.1010316 (PMC9321776; doi:10.1371/journal.pgen.1010316)

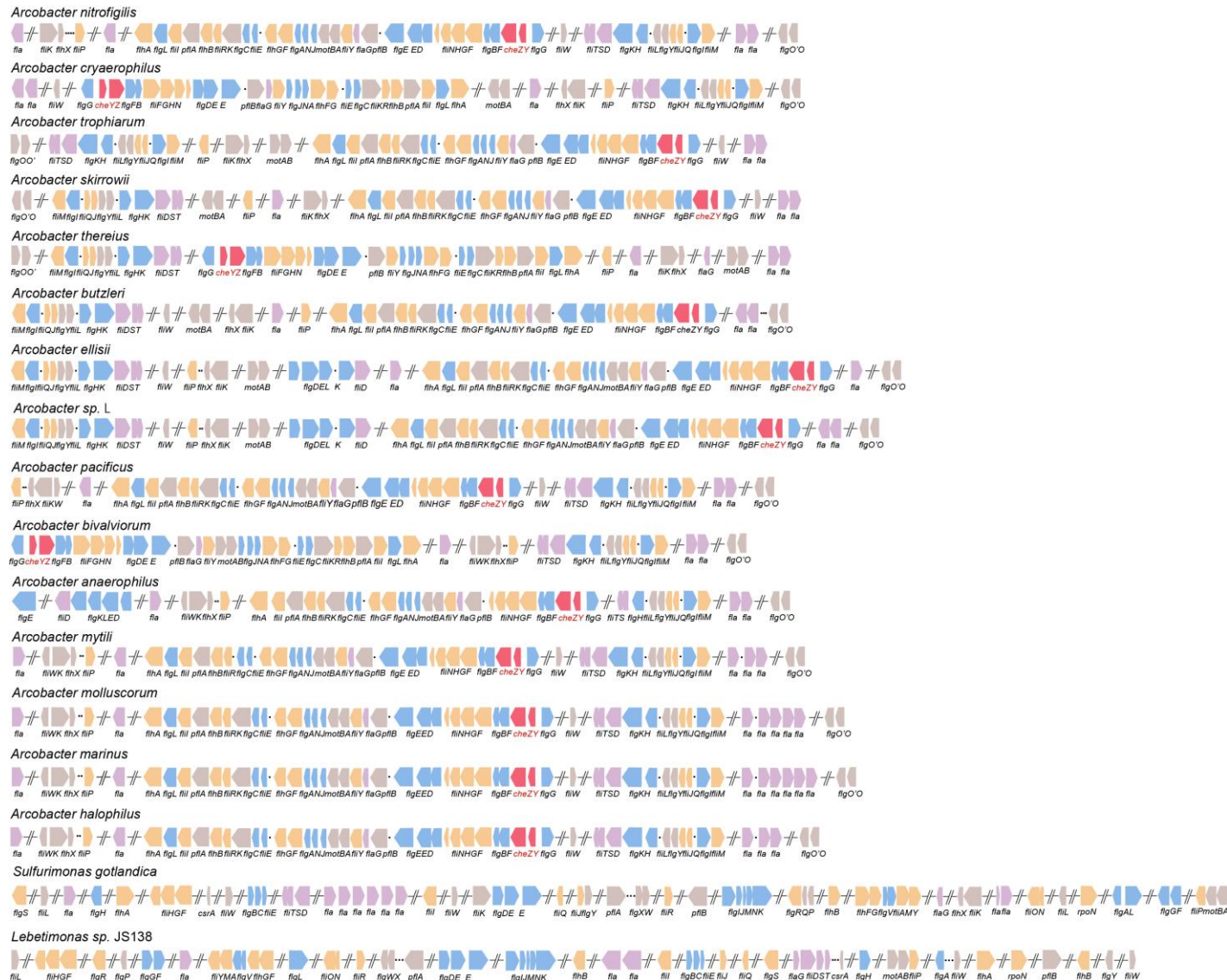

Supplement: S7 Fig — (PDF) [file pgen.1010316.s007.pdf]

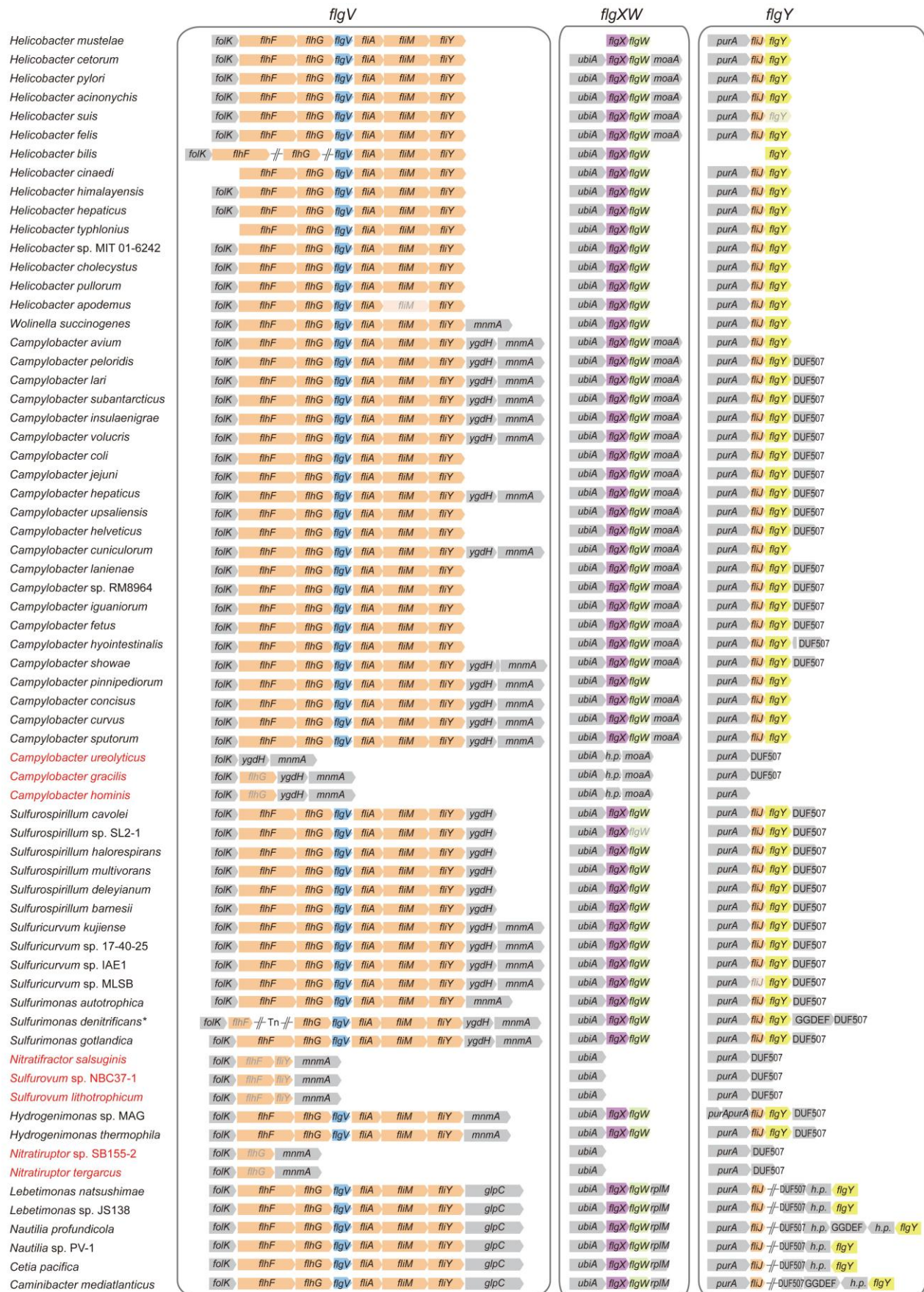

Supplement: S8 Fig — (PDF) [file pgen.1010316.s008.pdf]

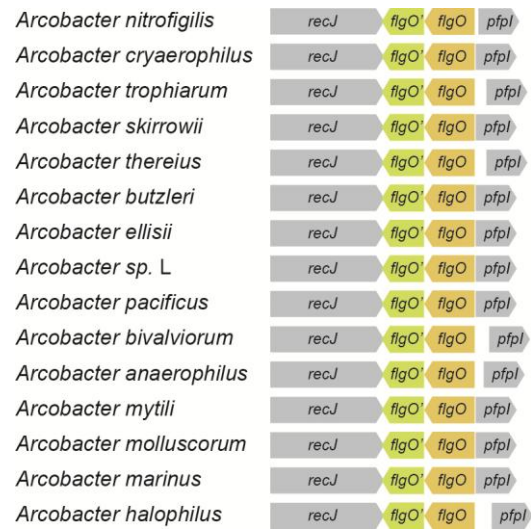

**S9 Fig.** The conserved gene order of *flgO* and its potential paralog *flgO'* in *Arcobacter* genomes.

Supplement: S9 Fig — (PDF) [file pgen.1010316.s009.pdf]

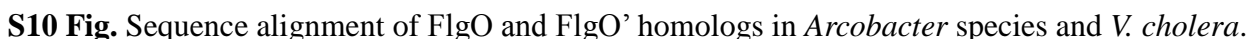

Supplement: S10 Fig — (PDF) [file pgen.1010316.s010.pdf]
