# Supplementary material for: The evolutionary path of chemosensory and flagellar macromolecular machines in Campylobacterota
Source: PLoS Genet. 2022 Jul 14;18(7):e1010316. doi: 10.1371/journal.pgen.1010316 (PMC9321776; doi:10.1371/journal.pgen.1010316)
Supplement: S2 Fig — (PDF) [file pgen.1010316.s002.pdf]

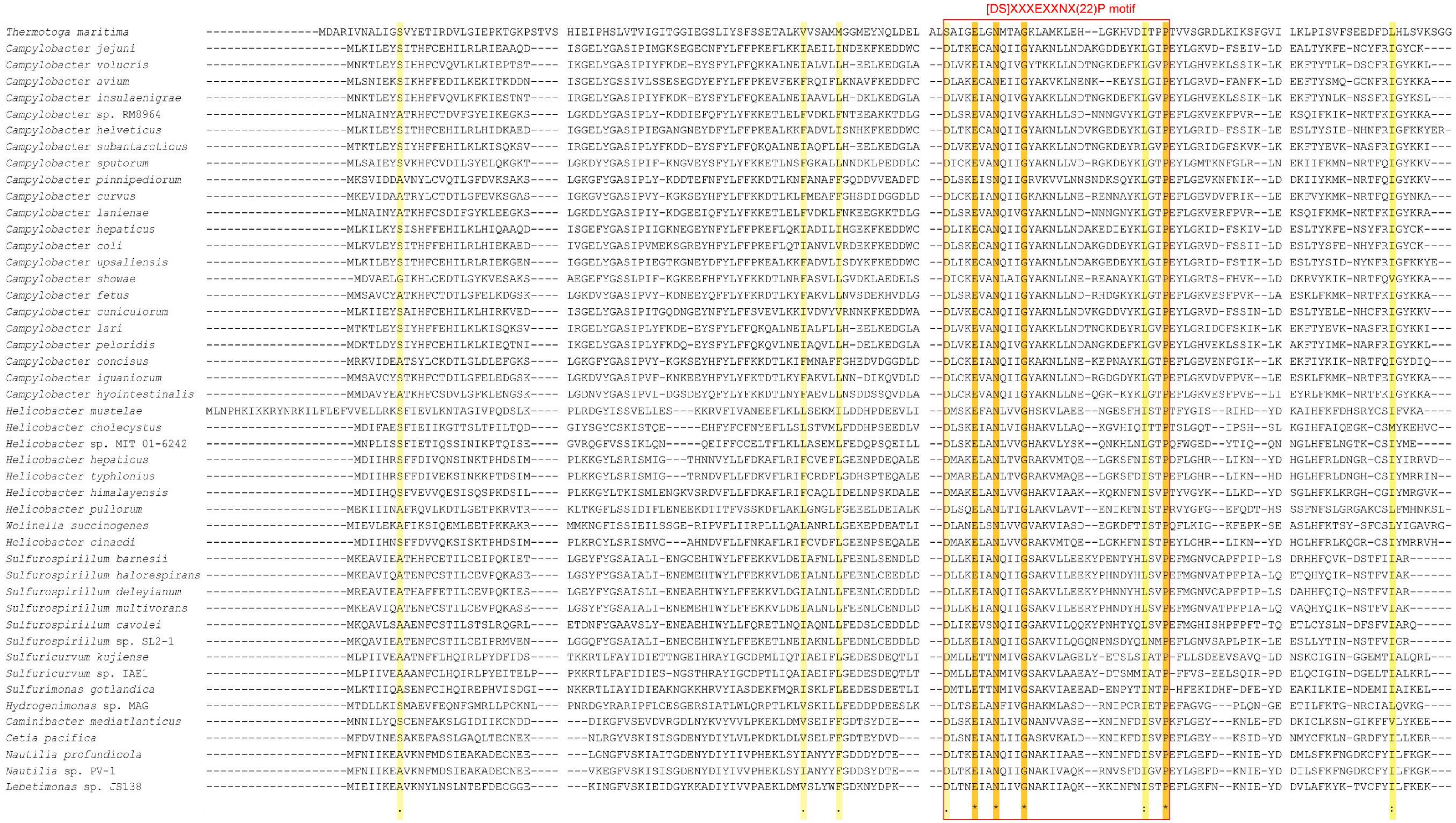

S2 Fig. Sequence alignment of potential CheX homologs in *Campylobacterota* species with experimentally confirmed CheX from *Thermotoga maritima* shown on the top.
