# Supplementary material for: The evolutionary path of chemosensory and flagellar macromolecular machines in Campylobacterota
Source: PLoS Genet. 2022 Jul 14;18(7):e1010316. doi: 10.1371/journal.pgen.1010316 (PMC9321776; doi:10.1371/journal.pgen.1010316)
Supplement: S3 Fig — (PDF) [file pgen.1010316.s003.pdf]

```
Secondary structure predicted by PSIPRED      ChHHHHHHHHHHHHHHhCCeeEeccC------CCCCeEEEEEEEecCCCCeEEEEEEEcHHHHHHHHH-HHcCCCC-CCh
C. jejuni  CheX                            1  MLKILEYSITHFCEHILRLRIEAAQD------ISGELYGASIPIMGKSEGECNFYLFFPKEFLKKIAE-ILINDEK-FKE 72 (140)
Consensus                                  1  ~~~~~~~s~~~~~~~~~~~~~~~~~~------~~~~~~~~~i~~~g~~~~~g~~~l~~~~~~~~~l~~-~~~g~~~-~~~ 72 (140)
                                              +.+.+.+++.++|+++++.+++....      .....+.+.+.+.|+.  +|.+.+.++++.+.++++ .|.|.+. +.+
Consensus                                  5  ~~~~~~~~~~~~l~~~~~~~v~~~~~~~~~~~~~~~~~~~~~~~~~~G~~--~G~~~l~~~~~~~~~i~~~~~~g~~~~~~~ 82 (155)
T. maritima CheX(1SQU_B)                   5  IVNALIGSVYETIRDVLGIEPKTGKPSTVSHIEIPHSLVTVIGITGGI--EGSLIYSFSSETALKVVSAMMGGMEYNQLD 82 (155)
Secondary structure                           HHHHHHHHHHHHHHHHhCCCCeeCCccccccCCCCceEEEEEEeeccc--EEEEEEEeCHHHHHHHHHHHhCCCCccccC

Secondary structure predicted by PSIPRED      hHHHHHHHHHHHHHHhHHHhhccccCCCCCcEEeeCCeEeCCCCc---ccceeceeEEEEECC-EEEEEEEe
C. jejuni  CheX                           73  DDWCDLTKECANQIIGYAKNLLNDAKGDDEYKLGIPEYLGKVDF---SEIVLDEALTYKFEN-CYFRIGYC 139 (140)
Consensus                                 73  ~~~~D~~~El~Nii~G~~~~~l~~~~~~~~~~~~~P~~~~~~~~---~~~~~~~~~~f~~~~-~~~~v~i~ 139 (140)
                                              ++..|+++|++|+++|++.+.+.+.+   ....+++|.+..+...   ..- ....++|.+++ +.+.+.+.
Consensus                                 83  ~~~~d~l~Ei~n~i~g~~~~~l~~~~--~~~~~~~P~~~~~~~~~~~~~~~~-~~~~i~f~~~~~g~~~~~~~ 150 (155)
T. maritima CheX(1SQU_B)                  83  ELALSAIGELGNMTAGKLAMKLEHLG--KHVDITPPTVVSGRDLKIKSFG-VILKLPISVFSEEDFDLHLS 150 (155)
Secondary structure                           HHHHHHHHHHHHHHHHHHHHHHHHHHcC--CceEecCCeEEecCCeEeeecC-cEEEEEeecCCCCceEEEEEE
```

S3 Fig. Pairwise sequence and secondary structural comparison of CheX from *Campylobacter jejuni* and *Thermotoga maritima*. The ([DS]xxxExxNx(22)P) motif is highlighted in red.
