# Supplementary material for: The evolutionary path of chemosensory and flagellar macromolecular machines in Campylobacterota
Source: PLoS Genet. 2022 Jul 14;18(7):e1010316. doi: 10.1371/journal.pgen.1010316 (PMC9321776; doi:10.1371/journal.pgen.1010316)
Supplement: Attachment — Submitted filename: Reply to Reviewers.pdf [file pgen.1010316.s027.pdf]

Reviewer #1: The manuscript by Mo et al performs a phylogenetic analysis on the Campylobacterota clade of bacteria and subsequently focus on the flagellar and chemotaxis homologs encoded in the genomes. I think they show that the most ancestral members of the clade have a flagellum whose genes are dispersed throughout the genome and a particular chemotaxis system type "F3". As one moves deeper in the tree, there are relatives in which all of the flagellar and chemotaxis genes are lost and later relatives reacquire a contiguous set of flagellar genes by horizontal transfer and the chemotaxis system that accompanies the change also is of a different type. There is an over-abundance of details presented, most of which meant essentially nothing to me, but the above is my take-home message (which may or may not be correct). The support for the flagellar substitution made sense to me but I really didn't understand the relevance of the different chemotaxis system types. Perhaps it would help to describe what goes into the definition of the different chemotaxis classes and why/how those classes are different or would matter (something about F9 being soluble but I didn't get much out of that). Maybe start with the idea that the flagellum changes and then describe the chemotaxis changes? As it stands now, the chemotaxis differences are presented first and later we learn the flagellum changes so the former makes sense because the chemotaxis systems are subordinate. Whatever the case, PLoS Genetics is a generalist journal and some greater effort should go into making the story generalist accessible, this is particularly important because I don't totally understand what is going on here and I'm a specialist in the field.

We thank the reviewer for pointing out insufficient background introduction and giving suggestions to restructure the manuscript. We made substantial revision through taking both reviewers #1 and #2's suggestions and making this manuscript readable for non-chemotaxis experts. Specifically for the above points, we made the following changes:

1. Explanation of different chemosensory classes was added in the introduction (Lines 74-80), supplemented with a **S1 Table** to show the details of differences among classes. The original definitions of chemosensory classes were proposed in 2010 based on sequence analyses, so we updated the information by adding structural features of F classes studied by Cryo-EM and additional differences such as cellular location, architecture or function.

2. Lines 80-90 explain why the diversity of chemosensory classes matters. Lines 519-527 emphasize why more studies need to be done for F9 class.

3. The theme of this paper is how chemosensory system evolved diversity such as F class switch or hybrid. To study this, we need to analyze the flagellar evolution together since they co-evolve. If we start with flagellar changes, it will be a different logic and requires more details in flagellar changes and corresponding changes not limited to chemosensory changes (motile/non-motile lifestyle changes many things). We are currently preparing another manuscript which focuses on flagellar evolution, its gain and loss in the genome context. We hope the reviewer understand why we focus on chemosensory diversity & evolution here. In addition, it's true that our last section in Results describing the evolution of high-torque complex flagellar structure might draw

attention. It is an exciting finding since we proposed different hypothesis regarding the evolution of complex motor, but this last point is not the main theme of this paper and please see more explanation in our reply to the last comment from Reviewer #2.

Line 54. “It” I assume the “it” is the flagellum but please clarify. Moreover the bacterial flagellum is rather narrowly distributed in the bacterial lineage and type IV pilus mediated twitching motility and divergent mechanisms of gliding are almost certainly more widespread than flagellar mediated movement. Finally, the archaellum of the archaea is not related to the bacterial flagellum. Reword.

We replaced “It” with “Chemosensory system”.

Line 59. “chemosensory system is evolutionarily dynamic in contrast to other highly conserved macromolecular machines” I don’t think this is true. I think the chemotaxis proteins are more highly conserved than the machines they control. For example, chemotaxis protein homologs control both the flagellum and the pilus of Pseudomonas aeruginosa and are more similar to each other than the flagella are to pili. Lines 77-79 support my concern.

We removed this sentence since Lines 72-90 with **S1 Table** better explain the diversity of chemosensory system now.

Para starting 128 seems like introductory material. Or rather, seems to contain no new information.

We moved this paragraph to “Introduction” now, see Lines 110-125.

Line 140 mentions CheA with an “additional receiver domain” but it is never mentioned how many receiver domains most CheA proteins have.

It was changed to “CheA protein with one additional receiver domain”, see Line 186 now.

To clarify: Most CheA protein doesn’t have any receiver domain, but F3-CheA, F5-CheA, ACF-CheA, and TFP-CheA have one receiver domain, please see the **S1 Table** for detailed information, which is adopted from Ref. 2.

Section starting 139. I don’t understand what this paragraph is trying to tell me. I think it is justification for calling the Campylobacter chemotaxis system an F3 system (I don’t know what this means either) but all of the information seems inferential and again previously reported. Perhaps better for introduction. Clarify the point of this paragraph. In general, I often found myself uncertain as to what information was new and what was previously published.

We thank the reviewer for constructive suggestions. The rationale for studying F3 is clarified in the first paragraph of Results, see Lines 151-155. We also changed the subtitle to “The conservation of F3 class in *Campylobacterota* is due to its primary role in flagellar motility”. Besides, some sentences as background information were moved to “Introduction”, such as Lines 130-134 now.

Line 158. Check syntax. The gene organization is described as unusual as they genes do not seem related but then the second clause says that it isn’t unusual to have genes organized like this? Clarify.

Yes, syntax can show the synteny of genomes and enable us to choose the genomes for display based on their phylogenetic relationship. What syntax shows is the same as our results in Fig. 1A and S1.

We confused our meaning by the word “However” in the original version. So we removed “However”, and it reads now: “The conserved gene orders of cheY-prmA, cheZ-trhP and cheX-fliNO in the *Campylobacterota* phylum are unusual because they are not functionally related, and it is known that the gene order is poorly conserved in bacteria [38, 39]. Their strict co-occurrence with the F3 class, especially in both deep-branching and later host-associated species that only have one F3 class, strongly support their association with this class.” (Lines 203-207).

To further clarify: as mentioned in Lines 187-190, it is difficult to identify CheY, CheZ and CheX for F3 class since they are dispersed from the core *cheVAW* operon (Fig. 1A). Especially for species with multiple F classes, it is even harder to discriminate their F class belongings and these species usually have many copies of CheY, some being phosphate sink, some functioning in two-component system rather than chemosensory system. We are surprised that we can locate F3-CheYZX by searching their downstream genes first.

Line 190. What is the H nomenclature? E.g. 36H, 44H etc?

Descriptions of H types were added in Lines 163-167. Accordingly, we modified **Fig. 1B** with details for the relationship of F classes and chemoreceptor H types.

Line 471. The statement suggests that Arcobacter specifically lost structural components but from my reading the manuscript, I thought the story was that all of the flagellar genes were lost and then Arcobacter reacquires a different complete set of flagellar genes by horizontal gene transfer. Thus, it didn’t actually lose certain (high torque) components in particular, it just reacquired a (low torque) flagellar system that doesn't require, and never had them.

We thank the reviewer for giving thoughts on the flagellar changes in *Arcobacter*, which is the most exciting puzzle to us. Please see our discussion in Lines 499-517, and we hope to bring attention for more research on *Arcobacter* flagellum.

Reviewer #2: This manuscript by Mo, et al reported the evolution of bacterial flagella and its chemosensory systems in the Campylobacterota phylum based on the sequence analysis. Although the manuscript presents a large amount of data, it is difficult to extract informative conclusions from the current dataset. As I described the below, the extent of this issue indicates that a simple revision may not be sufficient to raise the paper to the level required for publication.

We thank the reviewer for pointing out the writing problem and we made substantial revision in the current version.

A major concern is an apparent discrepancy between the results of this study and previously published work. The authors insist on analyzing the signal transduction system previously categorized as F3 class. The gene set of F3 class does not have the gene of cheX at the original definition in ref 2. However, this gene is including for their analyses in Fig 1 and elsewhere. I am concerned whether these analyses were performed correctly.

We thank the reviewer for pointing out CheX, which is a new finding in our study and not in the original definition of F3 class by ref. 2. Please see our description in Lines 196-198 and supporting information in S2 and S3 Fig that were newly added.

The Introduction part described a general discussion of other bacterial species, and there is little description of the bacteria belong to Campylobacteriota phylum. It is difficult to follow the line or arguments what is a fundamental breakthrough in the research area in this phylum.

Please see Lines 110-125 for description of the *Campylobacterota* phylum and our recent findings regarding chemosensory system in this phylum.

The first paragraph of Results and Discussion is a summery of ref 24, which is an authors' preprint in bioRxiv. It is unclear why the authors include this section as results.

We moved this paragraph to "Introduction", please see Lines 110-125.

Results and Discussions part is not always convincing and are often overinterpreted. Authors analyzed the flagellar genes in Nitratifractor and Arcobacterin Fig 2-5, but the motility and chemotaxis of these bacteria have not been well understood whether these gene are functional. The authors' conclusions are not well-founded.

We have done a thorough literature search regarding the presence of flagellar structure by EM imaging and the motile ability for all *Campylobacterota* species that were included in this study. Please see newly added **S2 Table** for information summarized from references that first isolated these species. Basically, two lineages are unflagellated and do not have flagellar and chemosensory genes, including

Sulfurovum/*Nitratifractor* genera and a subclade of the *Campylobacter* genus (please see Lines 179-182). Then, except that very few species whose genomes are assembled from metagenome lack flagellar/motility evidence, all the other species of this phylum are flagellated and motile according to published data. Particularly, for *Nitratiruptor* (I think the reviewer means *Nitratiruptor* not *Nitratifractor*) and *Arcobacter* in Fig 2-5, 14 of 15 *Arcobacter* species were reported to be motile, and 2 of 2 *Nitratiruptor* species were reported to be motile and flagellated (**S2 Table**).

The data presented here are merely correlative and do not show causal relationship for the flagellar structure and its torque generation. The authors' data are just sequence analyses. The precise measurements of the torque of flagellar motors are only restricted in few species, and the similar discussion about the motor structure have been already reported in ref 52. In addition, the images in Fig 6B are not original data of the author. This is a diversion of another authors' work from Movie S2 in ref 53, and Fig 2 in ref 26.

We agree with the reviewer that torque measurements are only done in few species, so we changed "high-torque motor structure" to "complex motor structure" for F3-containing *Campylobacterota* species, except for descriptions of *C. jejuni* and *H. pylori* that have been experimentally characterized for high torque. Although the motor structures of only 4 species of this phylum have been visualized by Cryo-EM as shown in Fig 6B, the presence of homologs of FlgPQ/PflAB (structural scaffolds), FliY with FliMN (wider rotor rings), FlgX (stator chaperon), FlgVWY (flagellar proteins with unknown function identified in *C. jejuni*) and the same set of regulators RpoN/FlgRS/FliA-FlgM/CsrA-FliW in all F3-containing *Campylobacterota* species suggest that these species most likely have similar complex structures as *C. jejuni, H. pylori* and *W. succinogenes* with the same flagellar gene composition, distinct from the model organism *E.coli* that lacks the above components. As a proof, *Arcobacter* species that belong to this phylum but lack these components display a different and simpler structure.

Of course, more Cryo-EM investigation of environmental *Campylobacterota* species, particularly the species from deep-sea hydrothermal vent as the ancestral lineage, will produce the ultimate proof of complexity in their motor structures. However, these species are anaerobic autotrophies that are very hard to culture in the laboratory. Also, they might not be amenable for Cryo-EM analyses that require the sample no thicker than a few hundred nanometers, whereas *C. jejuni* and *H. pylori* are perfect in this regard thus well studied. Therefore, bottlenecks exist for experimental characterization of non-model environmental species, but our comparative genomic studies provide useful information in the origin, conservation and diversity of flagellar motors of a fascinating phylum to facilitate functional studies.

Yes, previous ref 52 and 26, now ref 56 and 32 by Dr. Morgan Beeby's research group reported the Cryo-EM images of flagellar motors of *C. jejuni, H. pylori*, *W. succinogenes* and *Arcobacter butzleri,* and we adopted their images with citations. But

our conclusion regarding the evolutionary origin and direction of complex motor is the opposite to Dr. Beeby's viewpoint. Structural biologists are good at comparing different structures to link them together, and they assume that all complex structures evolve incrementally from simple ones. But evolution may not go this only direction. With no logic reason or evidence, Ref 32 and 56 picked up the simplest structure from *E. coli* as the starting point of flagellar evolution. But the flagellar genes in *E. coli* are HGT product from β-proteobacteria (see ref 46). Besides, they made wrong conclusion regarding the evolutionary order of different flagellar structure based on protein tree NOT species tree (Fig. 1 not Fig. 2 of ref 32 as the reviewer cited). Here, we used robust species tree (based on 92 single copy orthologs) to infer the branching order of different genera, and based on this order to track the evolutionary path of flagellar genes. In addition, we analyzed 8 more flagellar structural components (FliMNY, FlgO', FlgVWXY) and 7 regulators (RpoN, FlgRS, FliA-FliM, CsrA-FliW) (Fig. 6A), in addition to the 5 proteins (FlgOPQ, PlfAB) from ref 32. As the reviewer #3 pointed out that our analysis is "thorough and technically sound". Finally, our conclusions are different from Ref 32 and 56, see Lines 530-533: "Our analyses within the *Campylobacterota* phylum suggest that complex motors likely evolved in the common ancestor of this phylum. Later lineages with simpler motor structures, such as *Arcobacter*, lost some structural components and their motors were not the primitive form for this phylum."

The current manuscript is chemosensory-centric, we cannot elaborate more on the complex motor evolution since both reviewers #1 and #2 pointed out that we have "a large amount of data". The diversity and complexity of flagellar motors was revealed by Cryo-EM & cross-species comparison in 2011 (EMBO J, 2011), but the evolution of complex motors are not systematically studied since then except recent papers from Dr. Beeby. It is an important question and we would like to thoroughly discuss our viewpoints in a separate short article. We will present our analyses of flagellar motor structures in major bacterial lineages beyond *Campylobacterota*, showing the branching orders that we are confident with, and also pointing out current bottlenecks and future perspectives regarding this issue. If it is reasonable for the editor, we would like to submit to *PLOS Genetics* to be considered as an accompany Perspective to this manuscript.

Reviewer #3: This paper describes a thorough and technically sound analysis of chemosensory and flagellar systems in Campylobacterota, one of the many bacterial phyla.

Concerns:

1. English must be improved.

We have carefully checked grammars and improved the manuscript.

2. Multiple sequence alignments (e.g. in Fasta format) and phylogenetic trees (e.g. in Newick format) must be provided as supplementary files. At least the key ones.

We added additional supplementary files for sequence alignments and tree files:

S2 and S3 Fig for sequence alignments of CheX homolog;
S1 Data. Phylogenetic tree in Fig 1A, 2A and 6A in Newick format.
S2 Data. Phylogenetic tree in Fig 1C in Newick format.
S3 Data. Phylogenetic tree in Fig 2B in Newick format.
S4 Data. Phylogenetic tree in Fig 4B in Newick format.
S5 Data. Phylogenetic tree in Fig 5A in Newick format.
S6 Data. Phylogenetic tree in Fig 5C in Newick format.
S7 Data. Multiple sequence alignments of CheY homologs in Fig 5B in Fasta format.

3. Line 494: How the representative set of 2,781 species was selected? I cannot find information about this set anywhere in the supplement.

We downloaded the genomes of all representative species from NCBI Database on Mar 9th, 2021, when the list of representative species is 2781. We added this explanation in Lines 553-556 of Method section.
